# A Correlative Study Between Personality Traits and the Preference of Site Selection in Cosmetic Treatment

**DOI:** 10.3389/fpsyt.2021.648751

**Published:** 2021-05-19

**Authors:** Huan Qian, Yuxiao Ling, Chen Wang, Cameron Lenahan, Mengwen Zhang, Min Zheng, Anwen Shao

**Affiliations:** ^1^Department of Plastic Surgery, The Second Affiliated Hospital, School of Medicine, Zhejiang University, Hangzhou, China; ^2^School of Public Health, Hangzhou Medical College, Hangzhou, China; ^3^Center for Neuroscience Research, Loma Linda University School of Medicine, Loma Linda, CA, United States; ^4^Burrell College of Osteopathic Medicine, Las Cruces, NM, United States; ^5^Department of Dermatology, The Second Affiliated Hospital, School of Medicine, Zhejiang University, Hangzhou, China; ^6^Department of Neurosurgery, The Second Affiliated Hospital, School of Medicine, Zhejiang University, Hangzhou, China

**Keywords:** personality traits, cosmetic, personality questionnaire, rating scale, surgery

## Abstract

**Background:** Cosmetic treatment was closely associated with beauty seekers' psychological well-being. Patients who seek cosmetic surgery often show anxiety. Nevertheless, not much is known regarding how personality traits relate to the selection of body parts that receive cosmetic treatment.

**Aims:** This study aims to investigate the correlation between personality traits and various selection sites for cosmetic treatment via Eysenck Personality Questionnaire (EPQ).

**Methods:** A cross-sectional approach was adopted to randomly recruited patients from a general hospital planning to undergo cosmetic treatments. All respondents completed the EPQ and provided their demographic information. The EPQ involves four scales: the extraversion (E), neuroticism (N), psychoticism (P), and lying scales (L). Psychological scales were evaluated to verify that people who selected different body sites for cosmetic intervention possessed different personality portraits.

**Results:** A total of 426 patients with an average age of 32.14 ± 8.06 were enrolled. Among them, 384 were females, accounting for more than 90% of patients. Five treatment sites were analyzed, including the body, eye, face contour, nose, and skin. Comparatively, patients with neuroticism were more likely to undergo and demand rhinoplasty (OR 1.15, 95% CI 1.07–1.24, *P* < 0.001). Face contour treatment was commonly associated with extraversion (OR 1.05, 95% CI 1.00–1.11, *P* = 0.044), psychoticism (OR 1.13, CI 1.03–1.25, *P* = 0.013), and neuroticism (OR 1.05, CI 1.01–1.10, *P* = 0.019).

**Conclusions:** This novel study attempted to determine the personality profiles of beauty seekers. The corresponding assessments may provide references for clinical treatment options and enhance postoperative satisfaction for both practitioners and patients.

## Introduction

The pursuit of physical beauty is on the rise (1). Cosmetic treatment refers to modifications made to the human body's appearance in the absence of disease, injury, wound, congenital, or hereditary deformity, while also improving the quality of life (2). According to the American Society for Aesthetic Plastic Surgery (ASAPS), Americans splurged over $15 billion on cosmetic procedures in 2016 (3). In 2018, the total number of surgical and non-surgical procedures surged to 21.5 and 30.5%, respectively, over the last 5 years (4). The International Society of Aesthetic Plastic Surgery (ISAPS) ranked China second in this specific growth trend (5). As the demand for aesthetic procedures grows, people wish to transform their physical appearance to improve their psychological profile and psychosocial well-being (6).

People undergoing plastic surgery are not traditional patients with physical health issues. Psychological factors greatly motivate them to pursue surgery. Previous studies pertaining to aesthetic psychology mainly focused on cosmetic patients suffering from mental illnesses. Body dysmorphic disorder (BDD) is among the three most common psychiatric disorders in cosmetic patients (7). BDD patients are especially sensitive to minor flaws in their appearance (8) and demonstrate a tendency for dissatisfaction with surgical outcomes (9). Another common observation by past studies is borderline personality disorder (BPD), which was linked to emotional imbalance, impulsiveness, and self-image issues (10). These patients relentlessly demand and seek cosmetic surgery for self-injury. Hence, surgical treatment is best avoided in these patients (11).

Personality disorders are founded on the scientific principles of personality traits (12). However, the normal personality profiles in an average population are underreported. Here, some personality aspects of the general population seeking cosmetic treatments are analyzed. Various reports were linked with neuroticism and negative personality aspects; neuroticism is the most common personality trait in rhinoplasty patients (13). Perception of attractiveness increase in these rhinoplasty patients, improving their evaluation of their own attractiveness (14). Neuroticism is closely related to depression as a stable and heritable personality trait (15). Depression was also commonly seen in patients seeking aesthetic therapies. For example, patients who received breast implants exhibited high rates of suicidal ideation (16). Conversely, in other patients, breast augmentation effectively improved one's self-assessment (17). The negative correlation between cosmetic improvement and the intention in surgery suggested that cosmetic treatment helps increase life satisfaction.

Personality is described as a way of perceiving and relating the environment to oneself, and is affected by both genetics and the acquired environment. Relevant aspects of personality exist in a wide range of contexts, and are relatively stable over time (18). According to Eysenck, personality encompasses three major dimensions. Extraversion refers to sociability, vivacity, enthusiasm, and impulsivity. Neuroticism epitomizes depression, anxiousness, and emotional instability. Psychoticism signifies solitude, coldness, aggressiveness, and egocentricity (19). There exists an inadequate number of reports concerning the assessment of personality traits in patients prior to undergoing cosmetic procedures. Participants possessing psychopathological traits in the cosmetic industry were lower than expected, and their levels of anxiety did not cause dysfunction (20). Integral personality scales have rarely been applied to elucidate aesthetic orientation. How can we provide treatment options that match one's personality?

It is necessary to explore the association between personality profiles and the decision made in undergoing cosmetic treatment. Differences in personality lead to diverse choices, which are significant references for doctors and beauty seekers regarding recovery period, postoperative expectations, and design of the operation. Moreover, compared to the widely consumption beauty markets, cosmetology psychology in China is still in its infancy. In terms of plastic surgery, this study is the first to conduct personality questionnaires. It is hypothesized that different personality traits will influence the selection of treatment sites. This study may also assist in the clarification of psychological profiles, how they relate to an individuals' aesthetic tendency, and may contribute to optimal outcomes in therapy.

## Materials and Methods

### Participants

This was an observational cross-sectional study conducted at the Second Affiliated Hospital of Zhejiang University between September 1 and December 6, 2019. The inclusion criteria were: (1) participants were capable of filling out questionnaires independently; (2) participants demonstrated physical fitness without evidence of deformities, scars, or severe systemic diseases. Participants completed a demographic self-questionnaire with their name, gender, age, height, weight, income, marital status, education, smoking and drinking history, sleep duration, type of cosmetic treatment, and psychological scales. A total of 473 patients were investigated by random sampling. Finally, 426 questionnaires were included in the analysis, excluding questionnaires that rejected the survey, provided incomplete questionnaire information or untrue information.

The study was approved by the hospital Ethics Committee. All patients were informed of the objectives of the study, and they provided their informed consent.

We use the term “cosmetic treatment,” which is inclusive of surgeries (e.g., blepharoplasty, rhinoplasty, and liposuction) and non-surgical treatments (e.g., Botulinum toxin or filler injections and lasers).

### Measures

The EPQ (an 88-item self-reporting scale revised in China) was utilized in this study due to its excellent reliability and validity, which involves four scales: the extraversion (E), neuroticism (N), psychoticism (P) and lying scales (L). The higher the score, the more likely the personality traits listed in the scale are shown.

Based on the total score that each participant received in each scale, the standard score [T = 50 + 10^*^(X–M)/SD] was obtained by conversion. M and SD refer to the mean and standard deviation of the original scores achieved by the normal groups, respectively. Depending on the levels of the internal and external propensity scales and the neuroticism scale, the participants in the study were split into four classic temperaments: sanguineous (extroverted, stable), choleric (extroverted, unstable), phlegmatic (introverted, stable), and melancholic (introverted, unstable) (19).

### Statistical Analysis

Based on the statistics from 2000 (21), the average normal population in each dimension was calculated using the total scores of males and females in each dimension (number^*^mean) divided by the total number of participants. Participants who had or were predisposed to cosmetic treatment were considered the case group, with the remainder being the control group.

The data were input into Epidata 3.1 software, and Stata 15.1 was used for statistical analysis. All EPQ scores and demographic data were analyzed using descriptive statistics. Age, body mass index (BMI), sleep duration, and EPQ scores were regarded as continuous variables, whereas the cosmetic treatment tendency, results, and demographic parameters were considered categorical variables. The continuous variables were indicated by their mean and standard deviation (SD), and categorical variables were expressed as numbers (*N*) and percentages (%). A One-sample *t*-test was conducted to analyze the differences in EPQ scores at different sites. Moreover, a binary logistic regression analysis was conducted to investigate the correlation between EPQ scores at different sites and the demographic variables. *P* < 0.05 was regarded as being statistically significant.

## Results

The general information of all participants is presented in Table 1. There were 426 eligible patients included in this study, which was comprised of 384 (90.14%) females and 42 (9.86%) males. The age of the sample group ranged from 17 to 64.5 years (Mean = 32.14, SD = 8.06). Additionally, 310 (72.77%) participants possessed a bachelor's degree or higher, and 213 (50%) were married. Over 60% of participants exhibited a BMI within the normal range.

**Table 1:** General information of the participants included (*n* = 426).

**Characteristics**	**Means or proportions**
Age (years, mean ± SD)	32.14 ± 8.06
**Gender**, ***n*** **(%)**	
Male	42 (9.86)
Female	384 (90.14)
**Education**, ***n*** **(%)**	
Bachelor degree or below	116 (27.23)
Bachelor degree or above	310 (72.77)
**BMI**, ***n*** **(%)**	
<18.5	108 (25.35)
18.5–24	288 (67.61)
≥24	30 (7.04)
**Marital**, ***n*** **(%)**	
Single	213 (50)
Married	213 (50)
**Income**, ***n*** **(%)**	
Stable	128 (30.05)
Unstable	298 (69.95)
**Smoke**, ***n*** **(%)**	
Current/ever	26 (6.10)
Never	400 (93.90)
**Drink**, ***n*** **(%)**	
Current/ever	44 (10.33)
Never	382 (89.67)
**Sleep duration**, ***n*** **(%)**	
<7 h	51 (11.97)
7–8 h	174 (40.85)
≥8 h	201 (47.18)
**Eysenck Personality Scale, mean** **±** **SD**	
E score	11.48 ± 3.34
P score	4.80 ± 2.32
N score	11.39 ± 5.63
L score	11.67 ± 3.40
**Sites**, ***n*** **(%)**	
Skin	189 (44.37)
Nose	36 (8.45)
Face contour	119 (27.93)
Eye	103 (24.18)
Body	41 (9.62)

*E, extraversion; N, neuroticism; P, psychoticism; L, lying*.

Of the 426 participants, the preferred treatment sites were the skin, eyes (including eyebrows and lacrimal sulcus area), nose, face contour, and body (including breasts, abdomen, leg, shoulder, and labia minora). Cosmetic treatments involved laser rejuvenation, blepharoplasty, rhinoplasty, breast augmentation, chin augmentation, liposuction, botulinum toxin, or filler injections.

**Figure 1:**
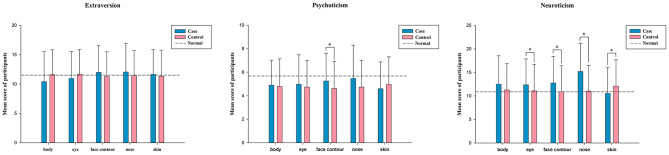
Distribution of EPQ scores in participants with or without cosmetic treatment according to different sites (=$\bar{\chi }$ ± SD). The case in the table refers to the participant who selects the site for cosmetic surgery, control refers to participants who have not selected the part for cosmetic surgery, normal refers to the average score of the general population in the scale. *The results are statistically significant (*P* < 0.05).

The mean and standard deviation values of the E score reached 11.48 ± 3.34, P score of 4.80 ± 2.32, N score of 11.39 ± 5.63, and L score of 11.67 ± 3.40, as shown in Table 1 and Figure 1 indicates the EPQ scores for participants with or without cosmetic treatment according to treatment sites. In the general population, the average of E was 11.50, while the averages of P, N, and L were 5.67, 10.88, and 12.56, respectively (21). Comparatively, three of the scales (E, P, and L) scored lower than the normal average, but N was higher than the normal average. As for L, lying signifies unsophisticated dissimulation, where participants scored lower than those in the normal population. A difference was found between participants with or without face contours in psychoticism (*P* for heterogeneity = 0.002). Moreover, there were significant differences regarding site location and neuroticism, such as in the eye (*P* = 0.045), face contour (*P* = 0.002), nose (*P* < 0.001), or skin (*P* = 0.004).

The association between the choice of cosmetic site and the EPQ scores for participants choosing to undergo treatment is depicted in Table 2. The model was adjusted with respect to age, gender, BMI, education, marital status, incomes, smoking and drinking history, and sleep duration. A statistically negative association was observed between skin treatment and N score risk (OR 0.96, 95% CI 0.92–1.00, *P* = 0.031). Those in the skin treatment group, which serves as an option in conventional medical rejuvenation, demonstrated lower levels of anxiety. A significant positive association was observed between cosmetic treatments performed on nose and N score risk (OR 1.15, 95% CI 1.07–1.24, *P* < 0.001), and a positive association was identified between face contour treatment and E, P, and N scores (OR 1.05, 95% CI 1.00–1.11, *P* = 0.044; OR 1.13, CI 1.03–1.25, *P* = 0.013; OR 1.05, CI 1.01–1.10, *P* = 0.019). In addition, regarding the four personality portraits, melancholic personality demonstrated a positive association with body treatment risk (OR 2.49, CI 1.21–5.12, *P* = 0.013). However, other personality traits were not statistically significant with respect to the choice in treatment sites.

**Table 2:** Association between cosmetic surgery choice and EPQ scores in participants according to different sites (*n* = 426).

	**Skin**		**Nose**		**Face contour**		**Eye**		**Body**	
	**OR (95% CI)**	***P***	**OR (95% CI)**	***P***	**OR (95% CI)**	***P***	**OR (95% CI)**	***P***	**OR (95% CI)**	***P***
E score	1.02 (0.98, 1.07)	0.370	1.01 (0.92, 1.10)	0.863	**1.05 (1.00, 1.11)**	**0.044**	0.95 (0.90, 1.01)	0.076	0.94 (0.87, 1.02)	0.131
P score	0.95 (0.87, 1.04)	0.285	1.11 (0.95, 1.29)	0.184	**1.13 (1.03, 1.25)**	**0.013**	1.03 (0.93, 1.14)	0.575	1.03 (0.89, 1.19)	0.717
N score	**0.96 (0.92, 1.00)**	**0.031**	**1.15 (1.07, 1.24)**	** <0.001**	**1.05 (1.01, 1.10)**	**0.019**	1.04 (1.00, 1.09)	0.068	1.05 (0.98, 1.12)	0.143
Phlegmatic	0.89 (0.19, 4.24)	0.888	–		–		2.40 (0.50, 11.46)	0.272	–	
Melancholic	0.76 (0.46, 1.27)	0.301	1.62 (0.68, 3.87)	0.276	0.88 (0.51, 1.55)	0.668	1.26 (0.71, 2.24)	0.432	**2.49 (1.21, 5.12)**	**0.013**
Sanguineous	1.60 (0.80, 3.20)	0.186	1.03 (0.28, 3.78)	0.961	0.67 (0.27, 1.62)	0.371	0.55 (0.22, 1.39)	0.206	0.88 (0.25, 3.08)	0.840
Choleric	1.02 (0.66, 1.57)	0.924	0.74 (0.34, 1.61)	0.445	1.40 (0.85, 2.32)	0.185	0.95 (0.57, 1.57)	0.846	0.53 (0.27, 1.05)	0.067

*OR, odds ratio; CI, confidence interval; E, extraversion; P, psychoticism; N, neuroticism*.

*Logistic regression adjusted for age, gender, BMI, education, marital status, income, smoking, drinking and sleep duration*.

*The values in bold mean that the results are statistically significant (P < 0.05)*.

**Figure 2:**
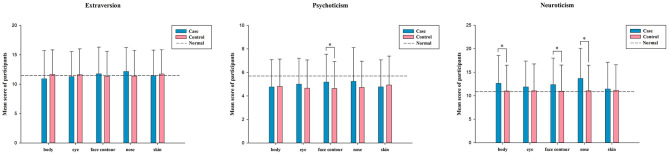
Distribution of EPQ scores in participants with cosmetic treatment tendency according to different sites (=$\bar{\chi }$ ± SD). The case in the table refers to the participant who prefers surgery on the site, control refers to participants who are not inclined to have surgery on that area, normal refers to the average score of the general population on the scale. *The results are statistically significant (*P* < 0.05).

The distribution of EPQ scores for participants with a tendency to receive cosmetic treatment according to different sites on their body is illustrated in Figure 2. This tendency suggests that participants wanted to achieve cosmetic transformation, but had yet to undergo an operation. Statistics showed that neuroticism held the highest mean EQP score, while psychoticism had the lowest mean score for different sites. A difference was discovered between the groups which with or without face contour treatment tendencies in those with psychoticism (*P* = 0.022). Additionally, there were significant differences existing in patients with neuroticism regarding body (*P* = 0.010), face contour (*P* = 0.017), or nose (*P* = 0.001) cosmetic treatment tendencies.

As shown in Table 3, an association is evident between the cosmetic treatment tendency and EPQ scores according to various sites. In terms of treatment tendency of cosmetic, the participants who preferred cosmetic surgery on nose (OR 1.07, CI 1.01–1.14, *P* = 0.018), eyes (OR 1.04, CI 1.00–1.08, *P* = 0.035) or body (OR 1.06, CI 1.01–1.10, *P* = 0.018) site had statistical difference in N score. Moreover, the tendency of eye surgery showed an association with the P score (OR 1.10, CI 1.00–1.20, *P* = 0.046). No significant differences were observed between the other scores and the four personality traits at different sites.

**Table 3:** Association between cosmetic surgery tendency and EPQ scores in participants according to different sites (*n* = 426).

	**Skin**		**Nose**		**Face contour**		**Eye**		**Body**	
	**OR (95% CI)**	***P***	**OR (95% CI)**	***P***	**OR (95% CI)**	***P***	**OR (95% CI)**	***P***	**OR (95% CI)**	***P***
E score	0.98 (0.92, 1.04)	0.452	1.03 (0.96, 1.11)	0.403	1.03 (0.98, 1.08)	0.241	0.98 (0.93, 1.02)	0.328	0.96 (0.91, 1.01)	0.135
P score	0.97 (0.87, 1.09)	0.663	1.06 (0.93, 1.20)	0.408	1.09 (0.99, 1.20)	0.067	**1.10 (1.00, 1.20)**	**0.046**	0.98 (0.89, 1.09)	0.770
N score	1.02 (0.97, 1.07)	0.534	**1.07 (1.01, 1.14)**	**0.018**	1.03 (0.99, 1.07)	0.212	**1.04 (1.00, 1.08)**	**0.035**	**1.06 (1.01, 1.10)**	**0.018**
Phlegmatic	–		1.42 (0.15, 13.39)	0.760	1.16 (0.20, 6.62)	0.865	1.02 (0.22, 4.81)	0.982	1.51 (0.27, 8.50)	0.638
Melancholic	1.23 (0.62, 2.44)	0.562	0.58 (0.25, 1.39)	0.224	0.92 (0.53, 1.58)	0.761	1.15 (0.70, 1.91)	0.578	1.46 (0.83, 2.55)	0.188
Sanguineous	0.87 (0.36, 2.13)	0.766	1.43 (0.51, 4.03)	0.499	0.84 (0.37, 1.90)	0.670	0.60 (0.29, 1.23)	0.161	0.65 (0.26, 1.63)	0.356
Choleric	0.81 (0.45, 1.44)	0.470	1.23 (0.62, 2.47)	0.554	1.12 (0.70, 1.81)	0.634	1.09 (0.71, 1.69)	0.687	0.83 (0.50, 1.37)	0.473

*OR, odds ratio; CI, confidence interval; E, extraversion; P, psychoticism; N, neuroticism*.

*Logistic regression adjusted for age, gender, BMI, education, marital status, income, smoking, drinking and sleep duration*.

*The values in bold mean that the results are statistically significant (P < 0.05)*.

## Discussion

To our knowledge, this is the first study employing EPQ to assist with the psychometric analysis of cosmetic treatment. Participant profiles were predominantly young females with a high level of education, healthy lifestyle, and insecure incomes. These findings were consistent with Wei's study in regard to the general information (22). In their study, young women were the main patients, including some high school and college students Women were more dissatisfied with their body compared to men (23). With the change in aesthetics and the influence of social media, women pay more attention to their personal appearance, physical dissatisfaction may have a negative impact on the patient's self-image, social status, employability and interpersonal relationships (24). Health conditions and depression also participate in encouraging people to have cosmetic surgery (25, 26). Additionally, public perception of cosmetic surgery has also changed as more people have become educated (1). Further studies have found that psychological factors, such as the pursuit of beauty and body satisfaction have a greater impact on cosmetic surgery patients than social factors, like having higher social standing and building good interpersonal relationships (27). We investigated the psychological characteristics to determine the treatment preferences of different groups in hopes of achieving improved treatment effects and outcomes.

The most popular surgical sites were the skin, face contour, and eyes in our study, which align with other findings (28). Patients <20-year-old were likely to choose minor interventions, such as skin treatment, while those over 45 preferred rejuvenation procedures (29). Moreover, as Chinese are more conservative regarding aesthetics, indications for surgery are also more conservative than in other countries (30), breast augmentation is less considered. In view of treatment choices, participants showed a distinguished personality profile. First, the inward- and outward-leaning personality types are the most prominent psychological types proposed by Carl Jung. This dimension relates to the intensity of excitability and inhibition of the central nervous system. Extroverts tend to have additional social needs, seek more social interaction and value their appearance to gain energy from their environment (31). Hence, they may be more motivated to attain beauty through medical procedures. Second, neuroticism was the most common psychological trait motivating patients to undergo cosmetic surgery on the nose, eyes, and other body parts (e.g., breast, legs and shoulders). Neuroticism may be represented by depression, anxiety, and emotional instability. Patients manifesting depressive symptoms often possess lower levels of self-esteem (32). They expect to achieve their desired body image and hope to reduce their anguish and dissatisfaction. Third, patients with a high degree of psychoticism scores have a potential psychopathological-associated personality trait. They are likely to feel lonely and ignore others, and they tend to exhibit peculiar behavior like suicide while disregarding danger (33). Therefore, when they consider undergoing plastic surgery, they may ignore the risks.

Among the patients who have undergone plastic surgery, neuroticism scores for the nose were the highest, which referred to its susceptibility for negative impacts, such as negative emotions (34). A high level of consistency was present where neurotic patients demonstrated interest in having nose treatments and followed through with receiving a rhinoplasty. According to Brucoli, rhinoseptoplasty patients are characterized by anxiety, depression, and less pronounced passivity, but exhibit higher levels of self-esteem (35). Patients who sought rhinoplasty for aesthetic motivations felt more depressed than those seeking functional rhinoplasty (36). In addition, obsessiveness and narcissism were detected in patients seeking rhinoplasty (13, 37). The human nose is considered the most prominent midline projection of the face (38), and is considered the most noticeable and concerning site regarding one's personal characteristics. Moreover, congenital defects or aesthetic needs of the nose may lead to dissatisfaction and negative long-term effects on self-esteem. Low levels of self-acceptance confer significant impacts on psychological resilience by causing anxiety and depression. This study confirmed the presence of a significant difference in the N score, which shows that the most attention was given to the nose. Patients who were satisfied with their rhinoplasty outcomes found that the procedure helped improve their body image and quality of life while boosting their self-confidence and self-esteem (39).

However, in those repeatedly dissatisfied with their surgical outcomes, consideration should be given to BDD patients (9). Identifying BPD patients or even those with psychotic personalities is necessary, as they may frequently demand treatments for multiple sites (11). They figuratively choose to attack the most prominent part of the face to alleviate the discomfort caused by their personality disorder. The EPQ questionnaire is limited to a screening questionnaire. Consideration can be given to the Adverse Childhood Experiences questionnaire and the Structured Interview of Personality Organization (STIPO) to carry out a more comprehensive screening.

Facial contour adjustment was indicative of extraversion, psychoticism, and neuroticism. Furthermore, the score of EPQ was higher than the control group. In previous studies, similar results were obtained. Participants with experience in masseter injections or mandibular therapies scored high in extraversion, agreeableness, openness, and neuroticism (40). These traits were often reflected in behavior rather than in psychoticism (37). Essentially, they resorted to cosmetic treatments due to a lack of confidence, which was associated with specific physical defects and the desire to socialize (41). In addition, the contour of the mandibular angle was significant to the facial shape of Asian women, who believe that women with wide and square faces were more likely to be unhappy (42). Therefore, these patients are obsessed with having oval shaped faces, smooth tapered jaws, and round, pointy chins following treatment (43).

In this study, participants who underwent treatment on certain body parts showed melancholic personality traits. Evidence showed that participants shifted from substantially alleviated depression following treatment, and from self-loathing t to self-appreciation, illustrating an improved outlook on life (44). Certain depressed patients shown psychosomatic manifestations, and they improved their self-esteem and depressive symptoms following cosmetic surgery (45).

Regarding the tendencies for cosmetic treatment, a significant difference existed in the N score of the treatment of body sites, which is likely delayed in actuality. For example, liposuction or breast augmentation may carry high risks and a lengthy recovery. Interestingly, the human eye is another focus on the face, indicating the personality traits of neuroticism and psychoticism as well. However, the eyes can be refined by the use of cosmetics, glasses, and other modifications. Therefore, no significant difference was observed in our final results.

Our study contains several limitations. First, the majority of participants were located in east China, which may cause selection bias. Second, male data (*n* = 42) was inadequate in comparison with that of females. Third, the EPQ self-reporting scale is the most commonly conducted personality test in China due to its convenient implementation. It reflects a relatively limited range of personality types, making it difficult to conduct a more precise personality assessment. The different dimensions of the personality questionnaire should be further expanded, such as the Cattell Sixteen Personality Factor or the Big Five Personality Inventory. Despite these limitations, this study's findings highlight the impact of personality traits on different sites of cosmetic surgeries. In the future, postoperative satisfaction should be assessed, and more screening procedures should be designed for clinical intervention to avoid unnecessary surgeries.

## Conclusion

The personality profile of participants receiving cosmetic treatment was more depression, anxiety, emotional instability and non-sophisticated. Different personality traits influence the site selection for cosmetic therapy. Physicians should consider neuroticism in patients seeking rhinoplasty. If experienced surgeons identify personality traits prior to undergoing cosmetic surgery, patients may benefit from better rehabilitation and increased satisfaction.

## Data Availability Statement

The raw data supporting the conclusions of this article will be made available by the authors, without undue reservation.

## Ethics Statement

The studies involving human participants were reviewed and approved by the Human and Research Ethics committees of the Second Hospital of Zhejiang University. Written informed consent to participate in this study was provided by the participants' legal guardian/next of kin. Written informed consent was obtained from the individual(s), and minor(s)' legal guardian/next of kin, for the publication of any potentially identifiable images or data included in this article.

## Author Contributions

AS and MZhe: conceptualization and methodology. HQ: writing- original draft preparation. YL: formal analysis. MZha: data curation. CW: investigation. CL: review and editing. All authors contributed to the article and approved the submitted version.

## Conflict of Interest

The authors declare that the research was conducted in the absence of any commercial or financial relationships that could be construed as a potential conflict of interest.
